# Preoperative Chemoradiotherapy with Tegafur-Uracil, Capecitabine, or 5-Fluorouracil/Leucovorin for Rectal Cancer in an Asian Cohort: A Real-World Comparison from the Pre-TNT Era

**DOI:** 10.3390/curroncol33020079

**Published:** 2026-01-30

**Authors:** Kun-Yao Dai, Fred Yi-Shueh Chen, Chien-Kuo Liu, Johnson Lin, Shih-Hua Liu

**Affiliations:** 1 Department of Radiation Oncology, MacKay Memorial Hospital, Taipei City 104217, Taiwan; 2 Department of Medicine, MacKay Medical University, New Taipei City 252005, Taiwan; 3 Department of Physical Medicine and Rehabilitation, Ditmanson Medical Foundation Chia-Yi Christian Hospital, Chiayi City 60002, Taiwan; 4 Institute of Traditional Medicine, School of Medicine, National Yang Ming Chiao Tung University, Taipei City 112304, Taiwan; 5 Division of Colorectal Surgery, Department of Surgery, MacKay Memorial Hospital, Taipei City 104217, Taiwan; crs.liuck@gmail.com; 6 Division of Hematology and Oncology, Department of Internal Medicine, MacKay Memorial Hospital, Taipei City 104217, Taiwan

**Keywords:** rectal cancer, chemoradiotherapy, tegafur-uracil, neoadjuvant therapy

## Abstract

Preoperative concurrent chemoradiotherapy (CCRT) is an important treatment for locally advanced rectal cancer, but the choice of chemotherapy utilized with radiotherapy is inconsistent. Guidelines mainly recommend 5-fluorouracil/leucovorin (5-FU/LV) or capecitabine, whereas tegafur-uracil (UFT) is widely used in Asia with limited comparative data. We evaluated UFT versus capecitabine and 5-FU/LV in an Asian real-world cohort. Between 2012 and 2019, 79 patients with biopsy-proven cT2–4N0–N2 rectal cancer received pelvic radiotherapy plus concurrent UFT (n = 31), capecitabine (n = 30), or 5-FU/LV (n = 18), followed by surgery. Endpoints included acute toxicity, pathologic complete response (pCR), T/N downstaging, overall survival (OS), and recurrence-free survival (RFS). Diarrhea was the most common toxicity (grade 1–2 in 68.4%). Neutropenia differed by regimen (UFT, 0%; capecitabine, 20.0%; 5-FU/LV, 16.7%), with one grade 3 event (5-FU/LV). The overall pCR rate was 17.7% (UFT, 16.1%; capecitabine, 23.3%; 5-FU/LV, 11.1%), and nodal downstaging was more frequent with capecitabine. After a median follow-up of 39.1 months, the 3-year OS and RFS were 88.9% and 68.9%, respectively, without significant survival differences among regimens. UFT-based long-course CCRT appears feasible and generally tolerable in routine Asian practice, with no clear signal of substantially worse pCR or survival outcomes in this retrospective cohort. These real-world data can inform individualized regimen selection.

## Simple Summary

Most clinical trials of preoperative chemoradiotherapy for rectal cancer have used only two chemotherapy drugs: capecitabine or 5-fluorouracil/leucovorin (5-FU/LV). In many Asian countries, another oral drug, tegafur-uracil (UFT), is widely used in daily practice but has been less well-studied in this setting. We reviewed 79 Asian patients with rectal cancer who all received radiotherapy before surgery together with one of three chemotherapy regimens: UFT, capecitabine, or 5-FU/LV. We compared acute side effects, pathologic complete responses, downstaging, and survival after treatment. All three regimens were generally well-tolerated and showed no obvious differences in major oncologic outcomes, while UFT tended to cause fewer problems with low white blood cell counts. Our findings suggest that UFT is a reasonable, convenient, and cost-conscious oral option for older or frail patients and for real-world practice in Asian settings.

## 1. Introduction

### 1.1. Background

Colorectal cancer (CRC) is the third most commonly diagnosed malignancy worldwide and ranks second in terms of mortality [1]. In many Asian countries, CRC incidence has risen steadily, driven by westernized diet, an aging population, smoking, physical inactivity, obesity, and other risk factors [2,3]. About one-third of CRCs arise in the rectum, and locally advanced rectal cancer (LARC) remains challenging because of substantial risks of local failure and distant metastasis.

For LARC, preoperative concurrent chemoradiotherapy (CCRT) followed by total mesorectal excision (TME) has long been the standard of care. The German CAO/ARO/AIO-94 trial established preoperative CCRT as superior to postoperative CCRT in terms of local control and toxicity. Preoperative CCRT entails the opportunity for downstaging, with some patients achieving a pathologic complete response (pCR), which allows sphincter preservation and enhancement of curative surgery [4,5]. On this backbone, fluoropyrimidine-based radiosensitization with intravenous 5-fluorouracil (5-FU) or oral capecitabine is recommended by major guidelines [6,7].

More recently, total neoadjuvant therapy (TNT), delivered as either induction chemotherapy followed by CCRT or CCRT followed by consolidation chemotherapy, has been adopted for patients with LARC [8]. TNT is supported by randomized trials showing higher pCR rates and improved distant controls compared to conventional CCRT followed by surgery and adjuvant chemotherapy [9,10,11,12,13,14,15,16]. Nevertheless, in both standard CCRT and TNT, concurrent chemotherapy remains predominantly fluoropyrimidine-based.

### 1.2. Rationale and Knowledge Gap

Oral tegafur-uracil (UFT) is another oral fluoropyrimidine active in CRC [17] and has been used more commonly in patients with gastrointestinal malignancies in Asia. UFT is composed of 100 mg tegafur and 224 mg uracil. Trials in metastatic and adjuvant CRC have demonstrated efficacy comparable to 5-FU/leucovorin (LV) and a favorable toxicity profile [18,19,20]. However, few studies have directly compared UFT, capecitabine, and 5-FU/LV as concurrent regimens in preoperative CCRT [21,22,23,24]. In an aging population with increasing frailty, oral treatment provides convenience in care and avoids catheter-related complications [25,26]. In Taiwan, regimen selection is further influenced by National Health Insurance reimbursement and patient economic burden; preoperative capecitabine often requires self-payment, whereas UFT is less expensive and more accessible.

### 1.3. Objective

Herein, we have retrospectively analyzed a single-institution cohort of patients with rectal cancer, treated with preoperative long-course CCRT between 2012 and 2019, before routine adoption of TNT. We have compared oral UFT, oral capecitabine, and intravenous 5-FU/LV as concurrent chemotherapy regimens with respect to pCR, downstaging, acute toxicities, and oncologic outcomes. This is a real-world evaluation aiming to clarify the potential role of UFT as a convenient and cost-conscious radiosensitizing option.

## 2. Materials and Methods

### 2.1. Patients and Eligibility

A total of 79 patients with rectal adenocarcinoma who received preoperative CCRT between January 2012 and December 2019 were selected for the present retrospective analysis. Patients were included for this study if they were diagnosed with localized rectal adenocarcinoma that was histologically proven. The inclusion criteria were as follows: (1) age 18 to 85 years old; (2) tumor located ≤12 cm from the anal verge; (3) clinically staged T3–T4 or N-positive or T2N0 low rectal cancer, defined as tumors with the inferior border within 5 cm of the anal verge, staged by colonoscopy, chest–abdominopelvic computed tomography (CT), or pelvic magnetic resonance imaging (MRI); (4) ECOG performance status of 0–1; and (5) ability to undergo surgical resection following preoperative CCRT. Patients who were diagnosed with a previous cancer, prior RT to the pelvic area, metastatic disease at diagnosis, or emergent operation during preoperative CCRT were excluded. All data, including survival and recurrence analyses, were calculated only up to 2 February 2021. This study was conducted in a single institution and approved by the institutional review board of the Mackay Memorial Hospital, Taipei, Taiwan (IRB No. 21MMHIS221e).

Pretreatment evaluation included medical history and physical examination, a digital rectal exam, complete blood count (CBC), serum chemistry, the carcinoembryonic antigen (CEA) level, a colonoscopy with biopsy, a chest X-ray, chest–abdominopelvic CT or pelvis MRI. All cases were restaged according to the AJCC 8th edition TNM system.

### 2.2. Preoperative Chemotherapy

All patients received pelvic radiotherapy (RT) with concurrent chemotherapy. In our institution, the choice of concurrent fluoropyrimidine regimen during long-course CCRT is individualized and determined by the treating physician in consultation with the patient, taking into account reimbursement status, clinical fitness, comorbidities and patient preference. The chemotherapy regimens were as follows: (1) intravenous therapy of 5-FU (400 mg/m^2^) and LV (20 mg/m^2^) for 5 days per week, administered at the first and fifth weeks of RT; (2) oral capecitabine prescribed at a dose of 1650 mg/m^2^/day, divided into two doses during the days of RT from Monday to Friday, with the weekend as a rest period; and (3) oral UFT (UFUR^®^; TTY Biopharm Co., Taipei City, Taiwan) prescribed according to the product label at a tegafur dose of 300–600 mg/day. At our institution, a total tegafur dose of 400 mg/day, administered continuously including weekends, is used as the routine starting dose, with subsequent stepwise reductions in 100 mg increments in the event of treatment-related toxicities.

Oral capecitabine is generally considered for clinically fit patients; however, in this indication, it was not reimbursed by Taiwan’s National Health Insurance (NHI) and therefore required full out-of-pocket payment, with an estimated cost of approximately USD 400–600 for a standard CCRT course. When this financial burden is prohibitive, intravenous 5-FU/LV, which is fully covered by NHI, is commonly selected as an alternative. UFT is typically prescribed for patients who refuse intravenous chemotherapy, are particularly concerned about toxicity, have more limited socioeconomic resources, have more comorbidities, or are older. UFT was given without LV modulation. Although combining UFT with LV may theoretically improve efficacy, the potential for increased toxicity has led our institution to adopt a conservative strategy of using UFT alone as the concurrent regimen. Chemotherapy was suspended and delayed if diarrhea or myelosuppression toxicities of grade 3 or worse occurred.

### 2.3. Radiotherapy

Patients were immobilized with an alpha cradle, and CT-based simulation was performed in the treatment position. The gross tumor volume (GTV) consisted of gross rectal tumor and pelvic lymphadenopathy. The clinical target volume (CTV) included the internal iliac lymph node below the L5–S1 spine level, the mesorectum, perirectal fat, and the presacral space up to the promontory. For T4 disease with adjacent organ involvement, external iliac nodes were also included. The planning target volume (PTV) was defined as the CTV plus a 0.8–1.0 cm margin. All patients received long-course pelvic RT. The PTV was prescribed 45 Gy in 25 fractions (1.8 Gy per fraction, five days per week). A boost to the primary tumor bed (GTV plus a 2 cm margin) was delivered to reach a total dose of 50–50.4 Gy, either as a simultaneous integrated boost (2 Gy per fraction) within the initial plan or as a sequential boost after repeat CT simulation near the 20th fraction. In patients who demonstrated near-complete regression of the primary lesion at replanning, the boost was omitted and the course was completed at 45 Gy. Treatment was delivered using intensity-modulated radiotherapy (IMRT) with five to seven fields with the planning software Eclipse Version 10 (Varian Medical Systems Inc., Palo Alto, CA, USA). The prescription dose was specified to the 100% isodose line, and plans were normalized to ensure that at least 95% of the PTV received 100% of the prescribed dose (PTV D95 ≥ 100%). RT was delivered using a 6–10 megavoltage linear accelerator. Details of dose constraints are provided in the Supplementary Materials.

### 2.4. Surgery

Patients were subjected to colonoscopy evaluation and restaged with abdominal–pelvic CT or MRI prior to surgery. Surgery was generally scheduled approximately 8 weeks after completion of preoperative CCRT. TME with either low anterior resection (LAR) or abdominoperineal resection (APR) selected as the surgical procedure was left to the discretion of the colorectal surgeon. Following surgery, the choice of postoperative adjuvant chemotherapy given at the discretion of the treating physician was not part of the study protocol.

### 2.5. Definition of Response

Tumor regression grades (TRGs) were graded according to the four-tier American Joint Committee on Cancer (AJCC)/College of American Pathologists (CAP) system [27], which assesses histologic treatment responses in the primary tumor bed, with the following categories: TRG 0, no viable residual carcinoma; TRG 1, rare single cells or small groups of residual carcinoma indicating near-complete regression; TRG 2, residual carcinoma with evident regression but more than scattered cells or small clusters; and TRG 3, extensive residual carcinoma with minimal or no histologic evidence of treatment response.

The pathological complete response (pCR) was defined as the complete absence of viable tumor cells (ypT0N0) in the surgical specimen. Downstaging was determined by comparing the pretreatment clinical T and N stages with the pathological T and N stages.

### 2.6. Adjuvant Chemotherapy

Adjuvant chemotherapy was not prespecified by protocol and was administered at the discretion of the treating oncologist according to patient age, comorbidities, pathological risk features, and preferences. Indications, regimens, and treatment duration were therefore heterogeneous and not standardized across regimens.

### 2.7. Follow-Up

All patients were followed up postoperatively every 3 months for the first 2 years. From the third to the fifth year, patients were followed every 6 months and annually thereafter. During follow-up, a chest X-ray, chest and abdominopelvic CT scans, and the CBC, including the CEA level, were performed.

### 2.8. Adverse Events Assessment

Patients were evaluated for toxicity by history taking, physical examination, and obtaining CBC and serum chemistry weekly during the periods of CCRT. Toxicity was graded according to the version 4.0 of the Common Toxicity Criteria for Adverse Events (CTCAE) published by the National Cancer Institute.

### 2.9. Statistical Analysis

All 79 patients were included in the efficacy and safety analyses. Baseline characteristics, treatment outcomes, and pathological findings were summarized for the three chemotherapy groups (UFT, capecitabine, and 5-FU/LV).

The primary endpoints were acute treatment-related toxicity and pCR rate. The secondary endpoints included (1) proportions of T and N downstaging; (2) overall survival (OS), which was defined as the time from the date of diagnostic biopsy to death from any cause, with patients alive at last follow-up censored on that date; and (3) recurrence-free survival (RFS), which was defined as the time from biopsy to the first documented local or distant recurrence or death from any cause, whichever occurred first, with patients without recurrence or death censored at the last disease assessment.

Continuous variables are presented as means ± standard deviation or medians with ranges. Between-group comparisons were performed using one-way analysis of variance (ANOVA). Categorical variables are reported as counts and percentages and were compared using the χ^2^ test or Fisher’s exact test when expected counts were small (<5). To estimate the association between chemotherapy regimen and pCR, we performed multivariable logistic regression, including chemotherapy regimen and a limited set of clinically relevant covariates [28]. Results were reported as adjusted odds ratios (ORs) with 95% confidence intervals (CIs). The Kaplan–Meier method was used to estimate OS and RFS, and 3-year survival rates with 95% CIs were reported. Survival curves across chemotherapy regimens were compared using the log-rank test.

To account for multiple comparisons within the family of the nine predefined acute toxicity endpoints, *p*-values were interpreted using a Bonferroni-adjusted significance level (overall α = 0.05; per-comparison α ≈ 0.0056). All tests were two-sided. For other efficacy and survival endpoints, *p* < 0.05 was considered statistically significant, and these analyses were prespecified as exploratory in this retrospective single-center study. Statistical analyses were performed using SPSS, version 22.0 (IBM Corp., Armonk, NY, USA), and survival curves were generated using the survival package of the R software (version 4.5.2; R Foundation for Statistical Computing, Vienna, Austria).

### 2.10. Generative Artificial Intelligence Tool

We used ChatGPT 5.1 (OpenAI, San Francisco, CA, USA) only to assist with language editing and refinement of this manuscript to comply with author guidelines and submission format requirements. This tool was applied after the study design, data collection, analysis, and interpretation had been developed and text content had been drafted by the authors. All suggestions were reviewed and verified by the authors, who take full responsibility for the content of this publication.

## 3. Results

A total of 79 patients treated between January 2012 and December 2019 were analyzed. There were 31 patients in the oral UFT group, 30 patients in the capecitabine treatment group, and 18 patients in the 5-FU/LV group. Their clinical and pathological characteristics are shown in Table 1. The majority of the patients were males (60.8%), and the median age was 59.0 years (range, 37–85 years). There were 47 patients (59.5%) with clinically node-positive disease (stage IIIA–IIIC). A total of 51 patients (64.6%) received surgery less than 8 weeks after preoperative CCRT. A total of 67 patients (84.8%) underwent LAR.

Overall, adjuvant chemotherapy was administered in 19/31 (61.3%) patients in the UFT group, 25/30 (83.3%) in the capecitabine group, and 13/18 (72.2%) in the 5-FU/LV group. In the UFT group, the most common regimen was oral UFT (12 patients). In the capecitabine group, oral UFT was the predominant adjuvant regimen (13 patients). In the 5-FU/LV group, the most frequently used adjuvant regimen was 12 cycles of FOLFOX (6 patients).

### 3.1. Toxicity

Chemoradiotherapy was generally well-tolerated with manageable side effects. Individual toxicities are shown in Table 2. The most common toxicity associated with preoperative CCRT was gastrointestinal toxicity. Of the 79 treated patients, 54 (68.4%) had grade 1–2 diarrhea. There were significant differences in neutropenia (UFT, 0%; capecitabine, 20.0%; 5-FU/LV, 16.7%; *p* = 0.003) and proctitis (UFT, 35.5%; capecitabine, 53.3%; 5-FU/LV, 11.1%; *p* = 0.004). One patient in the UFT treatment group experienced grade 3 radiation dermatitis, and one patient from the 5-FU/LV group had grade 3 neutropenia. Grade 4 anemia occurred in two patients, one in the UFT group and one in the 5-FU/LV group; both were managed with blood transfusion and temporary delay of chemotherapy.

### 3.2. Treatment Compliance

Treatment compliance with concurrent chemoradiotherapy was generally high across all groups. In the UFT group, one patient discontinued UFT prematurely and four patients required dose reduction. The median and mean daily tegafur doses actually delivered in the UFT group were 400 mg and 374 mg, respectively. In the capecitabine group, one patient discontinued capecitabine and four patients underwent dose reductions. In the 5-FU/LV group, one patient discontinued the second chemotherapy course. All patients completed the planned pelvic radiotherapy to the prescribed total dose; completion was delayed by more than one week in 3 patients in the UFT group, 1 patient in the capecitabine group, and 2 patients in the 5-FU/LV group. Overall, the relative dose intensity of concurrent chemoradiotherapy was broadly maintained across the three regimens.

### 3.3. Efficacy

**Figure 1:**
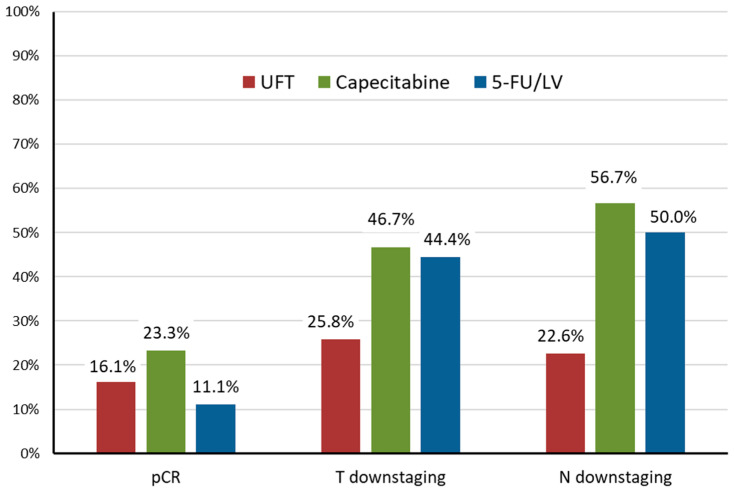
Proportions of patients achieving pathologic complete response (pCR), T-stage downstaging, and N-stage downstaging according to the concurrent chemotherapy regimen.

pCR was confirmed in 14 of 79 patients (17.7%). As shown in Figure 1, the pCR rates in the UFT, capecitabine, and 5-FU/LV groups were 16.1% (5/31), 23.3% (7/30), and 11.1% (2/18), respectively. In multivariable logistic regression adjusting for age, sex, clinical T stage, clinical N stage, and the interval between radiotherapy and surgery, neither capecitabine (adjusted OR, 1.21; 95% CI, 0.31–4.73; *p* = 0.788) nor 5-FU/LV (adjusted OR, 0.46; 95% CI, 0.07–3.02; *p* = 0.421) was significantly associated with higher odds of pCR compared to UFT (Table 3). Overall, 30 patients (38.0%) experienced T downstaging and 33 patients (41.8%) had N downstaging. Compared to UFT, capecitabine was associated with higher odds of N downstaging (crude OR, 4.48; 95% CI, 1.48–13.59; *p* = 0.008) (Table 3).

### 3.4. Survival

**Figure 2:**
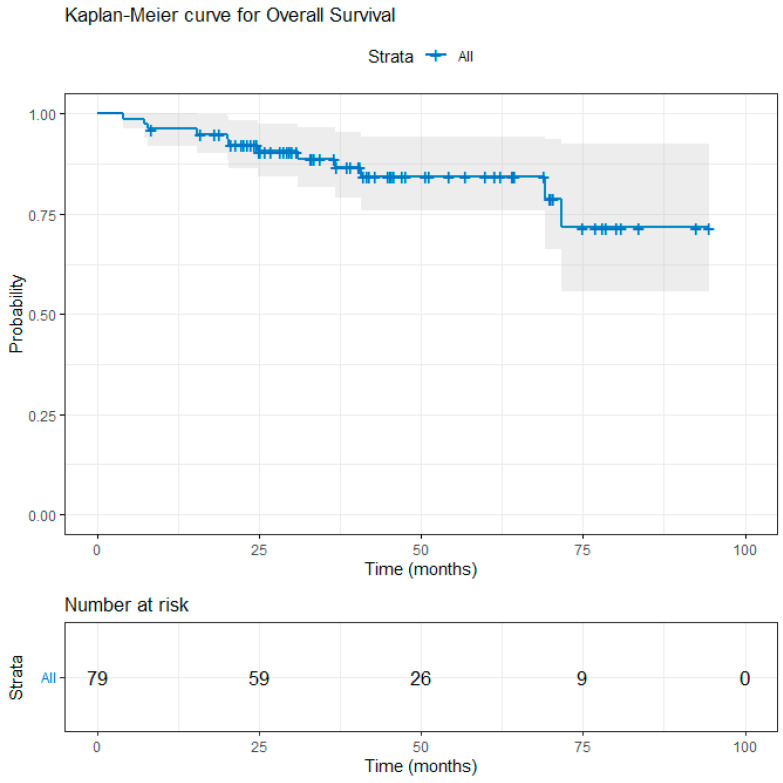
Kaplan–Meier curve for overall survival in the entire cohort. Vertical marks indicate censored observations. The estimated 3-year overall survival rate is 88.9% (95% CI: 81.8–96.6).

**Figure 3:**
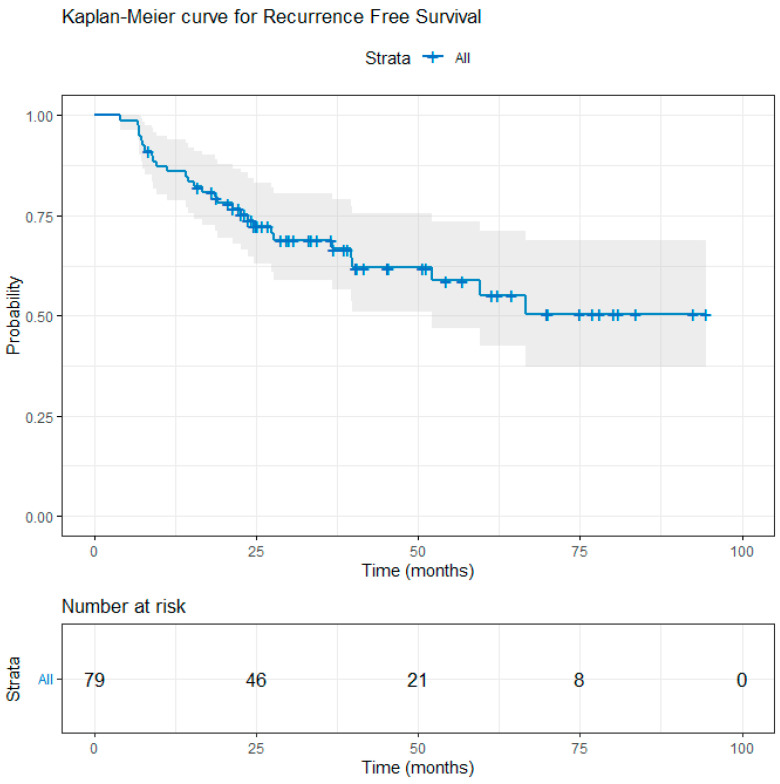
Kaplan–Meier curve for recurrence-free survival in the entire cohort. Vertical marks indicate censored observations. The estimated 3-year RFS rate is 68.9% (95% CI: 59.0–80.5).

**Figure 4:**
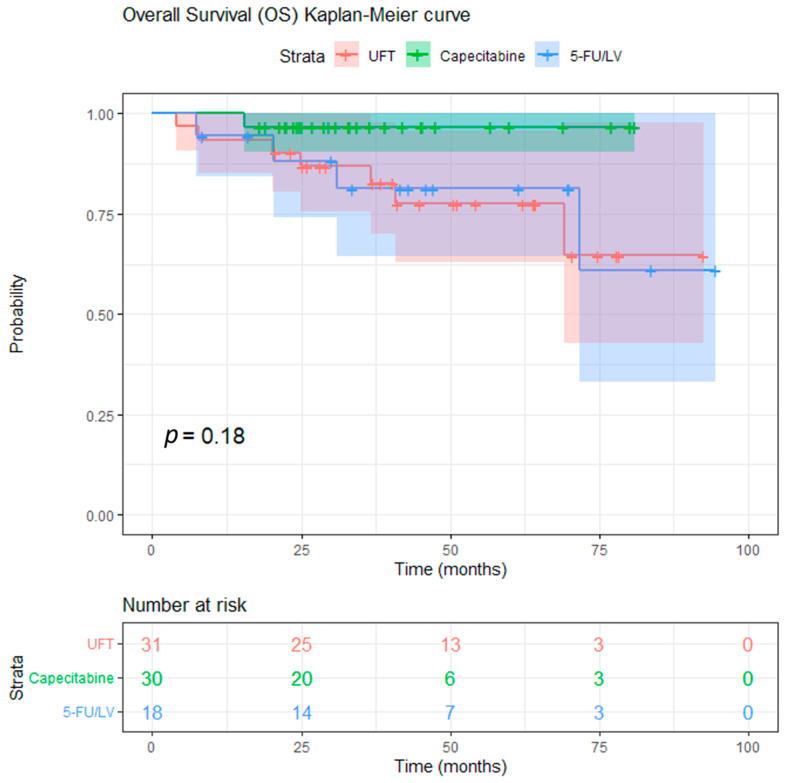
Kaplan–Meier curves for overall survival according to concurrent chemotherapy regimen (UFT, capecitabine, and 5-FU/leucovorin). Vertical marks indicate censored observations. The estimated 3-year overall survival rates (95% CI) are 86.8% (75.6–99.8) for the UFT group, 96.7% (90.5–100.0) for the capecitabine group, and 81.4% (64.3–100.0) for the 5-FU/leucovorin group.

**Figure 5:**
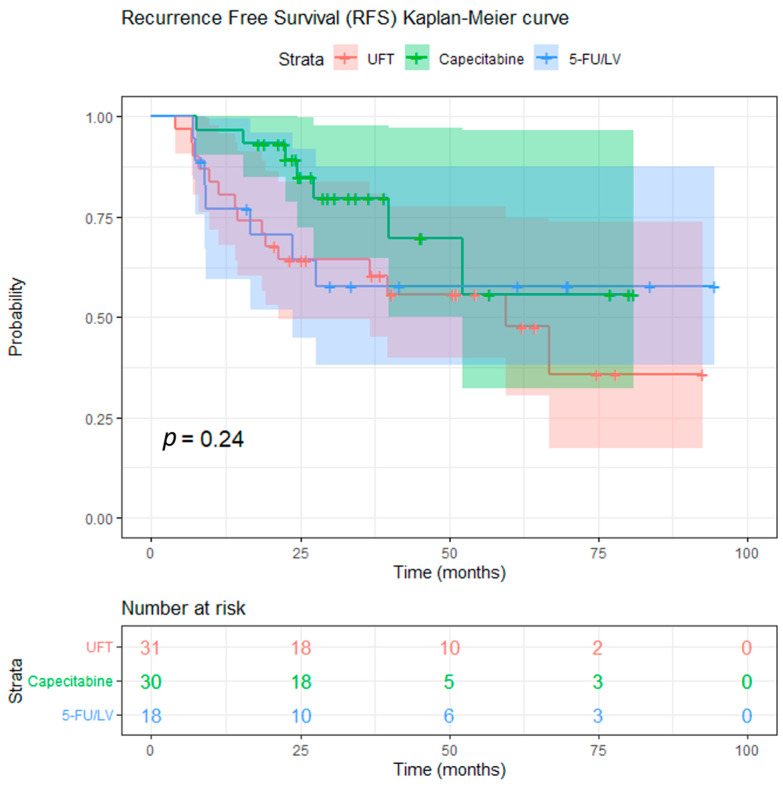
Kaplan–Meier curves for recurrence-free survival according to concurrent chemotherapy regimen (UFT, capecitabine, and 5-FU/leucovorin). Vertical marks indicate censored observations. The estimated 3-year RFS rates (95% CI) are 64.4% (49.5–83.7) for the UFT group, 79.7% (64.9–97.8) for the capecitabine group, and 57.8% (38.1–87.6) for the 5-FU/leucovorin group.

After a median follow-up of 39.1 months (range: 4.0–94.5 months), a total of 12 deaths were observed: 7/31 (22.6%) in the UFT group, 1/30 (3.3%) in the capecitabine group, and 4/18 (22.2%) in the 5-FU/LV group. Local recurrence occurred in 4 UFT-treated patients, 1 capecitabine-treated patient, and 1 patient in the 5-FU/LV group, whereas distant recurrence was documented in 8, 5, and 5 patients, respectively; additionally, one patient in the UFT group developed both local and distant recurrence. The 3-year estimated OS was 88.9% and the 3-year estimated RFS was 68.9% for the entire cohort (Figure 2 and Figure 3). There was no significant difference in OS and RFS between the three groups. The 3-year OS rates for the patients in the UFT, capecitabine, and 5-FU/LV treatment groups were 86.8%, 96.7%, and 81.4%, respectively (log-rank test, *p* = 0.074, UFT vs. capecitabine; *p* = 0.863, UFT vs. 5-FU/LV) (Figure 4). The 3-year RFS rates for the patients in the UFT, capecitabine, and 5-FU/LV treatment groups were 64.4%, 79.7%, and 57.8%, respectively (log-rank test, *p* = 0.112, UFT vs. capecitabine; *p* = 0.625, UFT vs. 5-FU/LV) (Figure 5).

## 4. Discussion

### 4.1. Key Findings

Of 79 patients with rectal cancer treated with preoperative long-course CCRT between 2012 and 2019, the overall pCR rate was 17.7%. The pCR rates in the UFT, capecitabine, and 5-FU/LV groups were 16.1%, 23.3%, and 11.1%, respectively, without statistically significant differences. Regarding oncologic outcomes, including RFS and OS, no statistically significant differences were observed among the three regimens. UFT had a favorable hematologic profile; no neutropenia was observed in this group, whereas grade 1–3 neutropenia occurred more often with capecitabine and 5-FU/LV.

### 4.2. Strengths and Limitations

This study has several strengths. First, it analyzed a clinically homogeneous cohort of patients with LARC, treated with standardized long-course CCRT and TME, using modern IMRT techniques and consistent RT protocols. Second, it provides a direct comparison of three commonly used fluoropyrimidine regimens as concurrent radiosensitizers, with all cases restaged according to the AJCC 8th edition and clearly defined oncologic endpoints. Finally, acute toxicities were systematically documented and graded according to CTCAE version 4.0.

This study has several limitations. It is a retrospective, single-institution analysis with relatively small sample and event numbers. Because regimen selection was strongly influenced by age, comorbidities, socioeconomic factors and reimbursement constraints, substantial confounding by indication is unavoidable. Accordingly, between-regimen comparisons should be interpreted as descriptive, hypothesis-generating real-world observations rather than evidence of therapeutic equivalence. Second, detailed MRI-based high-risk features (including circumferential resection margin involvement, extramural venous invasion, tumor length, depth of extramural spread, threatened sphincter or levator involvement, and nodal morphology) were not consistently and systematically reported in radiology records during 2012–2019. Therefore, these variables could not be reliably extracted and incorporated into our analyses. As a result, important determinants of response and prognosis may not have been fully captured, and residual confounding by baseline tumor biology and anatomic risk cannot be excluded. Third, the toxicity profile was limited by the retrospective nature of this study. Hand–foot syndrome, which is particularly relevant for capecitabine, and several other non-hematologic adverse events (e.g., mucositis, nausea/vomiting, fatigue), were not systematically recorded and may be under-represented in our dataset. Finally, adjuvant chemotherapy was not protocolized and varied by patient and physician preference, leading to heterogeneity in indications, regimens, and duration across groups, which may have influenced long-term outcomes.

### 4.3. Comparison with Similar Research

Our pCR rate and 3-year oncologic outcomes are consistent with previous reports of preoperative 5-FU based CCRT, where pCR rates of 8–25%, 3-year OSs of 75–92%, and 3-year RFSs of 65–76% have been described [4,21,24,29,30,31,32]. The high rate of nodal downstaging observed in the capecitabine group is in line with a phase 3 study revealing a trend of greater nodal downstaging with capecitabine compared to 5-FU [33].

In several recent TNT randomized studies, the standard CCRT arms reported pCR rates of approximately 12–14%, which is similar to the 17.7% observed in our cohort, whereas TNT regimens achieved better pCR rates, close to 28% [10,11,12].

UFT has been reported to be more tolerable in Japanese than in Caucasian populations in terms of gastrointestinal toxicity [34] and avoids hand–foot syndrome and diarrhea associated with capecitabine [33]. In this study, diarrhea was the most common adverse event and occurred in about two-thirds of patients, with more low-grade diarrhea and proctitis in the capecitabine group. In contrast, UFT was associated with no neutropenia, whereas the 5-FU/LV and capecitabine groups had more grade 2 or higher neutropenia and one case of grade 3 neutropenia. These observations are consistent with previous trials comparing UFT to 5-FU/LV, which reported fewer hematologic adverse effects for UFT-based regimens [18,21].

### 4.4. Explanations of Findings

The similar pCR rates observed across UFT, capecitabine and 5-FU/LV are consistent with the notion that these regimens share a common fluoropyrimidine backbone and might exert comparable radiosensitizing effects when delivered with adequate dose intensity. In contrast, the lower incidence of neutropenia in the UFT group likely reflects both pharmacologic differences and patient selection. UFT is associated with a more favorable hematologic toxicity profile in Asian populations. In our practice, UFT was often chosen for older or more comorbid patients who might not have tolerated intensive multi-agent chemotherapy. The absence of significant differences in survival endpoints between regimens probably reflects the small sample size limiting the study power and heterogeneity in postoperative systemic therapy and should not be interpreted as a proof of therapeutic equivalence.

### 4.5. Implications and Actions Needed

Chemotherapy with intravenous 5-FU/LV is time-consuming and sometimes requires hospitalization, which causes patient inconvenience. Oral fluoropyrimidines such as oral capecitabine or UFT are appealing alternatives that avoid venous access-related complications such as infection, thrombosis, and blockage [35]. Patients generally prefer oral chemotherapy, provided that efficacy is not compromised [36,37].

In the current era, TNT that incorporates oxaliplatin-based chemotherapy is recommended for patients with LARC. Nevertheless, a considerable proportion of patients in routine practice, particularly the very elderly, those with significant comorbidities, or those treated in resource-limited settings, still receive long-course CCRT without full TNT. Based on our real-world cohort, UFT-based CCRT yielded similar pCR and survival outcomes to capecitabine and 5-FU/LV, although capecitabine showed a stronger signal for nodal downstaging. Therefore, we position UFT as an alternative oral fluoropyrimidine option, particularly when intravenous infusional therapy is not feasible or when capecitabine is not suitable due to tolerability, availability, or cost considerations. Prospective studies are needed to clarify the role of UFT as a radiosensitizing backbone in both TNT and non-TNT strategies and to incorporate formal cost-effectiveness and patient-reported outcomes into treatment decision-making.

## 5. Conclusions

Given the range of available concurrent chemotherapy options, physicians need to refine the selection of appropriate preoperative CCRT for different patients. Our study showed that UFT-based long-course CCRT appears feasible and generally tolerable, with no clear signal of substantially worse pCR or survival outcomes. Differences in toxicity profiles and ease of administration may help inform chemotherapy selection for individualized patient care. This study provides additional real-world data that may help clinicians select concurrent chemotherapy regimens tailored to individual patient profiles in Asian clinical practice.

## Figures and Tables

**Table 1:** Patient and pathological characteristics.

	UFT (n = 31)	Capecitabine (n = 30)	5-FU/LV (n = 18)	Total (n = 79)	*p*-Value
Sex					0.821
Female	11 (35.5)	13 (43.3)	7 (38.9)	31 (39.2)	
Male	20 (64.5)	17 (56.7)	11 (61.1)	48 (60.8)	
Age (years)					0.106
Mean ± SD	61.6 ± 13.0	59.0 ± 9.9	54.5 ± 9.1	59.0 ± 11.3	
Median with range	60, 41–85	57.5, 37–76	56.5, 39–70	59, 37–85	
ECOG PS					0.259
0	24 (77.4)	26 (86.7)	12 (66.7)	62 (78.5)	
1	7 (22.6)	4 (13.3)	6 (33.3)	17 (21.5)	
Clinical T stage					0.730
T2	6 (19.4)	4 (13.3)	2 (11.1)	12 (15.2)	
T3	24 (77.4)	26 (86.7)	15 (83.3)	65 (82.3)	
T4	1 (3.2)	0 (0.0)	1 (5.6)	2 (2.5)	
Clinical N stage					0.082
N0	17 (54.8)	9 (30.0)	6 (33.3)	32 (40.5)	
N1	12 (38.7)	15 (50.0)	6 (33.3)	33 (41.8)	
N2	2 (6.5)	6 (20.0)	6 (33.3)	14 (17.7)	
Clinical TNM stage					0.142
I	3 (9.7)	1 (3.3)	1 (5.6)	5 (6.3)	
IIA	14 (45.2)	8 (26.7)	5 (27.8)	27 (34.2)	
IIIA	3 (9.7)	2 (6.7)	1 (5.6)	6 (7.6)	
IIIB	10 (32.3)	19 (63.3)	8 (44.4)	37 (46.8)	
IIIC	1 (3.2)	0 (0.0)	3 (16.7)	4 (5.1)	
CEA (ng/mL)	31.9 ± 66.8	7.7 ± 13.0	37.0 ± 95.4	23.9 ± 62.7	0.193
Tumor height (cm)					0.982
Mean ± SD	5.5 ± 3.3	5.5 ± 3.2	5.7 ± 3.5	5.6 ± 3.3	
RT-OP					
<8 weeks	20 (64.5)	20 (66.7)	11 (61.7)	51 (64.6)	0.927
≥8 weeks	11 (35.5)	10 (33.3)	7 (38.9)	28 (35.4)	
Mean ± SD	52.2 ± 21.4	53.1 ± 10.5	58.9 ± 23.5	54.0 ± 18.6	0.457
Surgical procedure					0.614
LAR	25 (80.6)	27 (90.0)	15 (83.3)	67 (84.8)	
APR	6 (19.4)	3 (10.0)	3 (16.7)	12 (15.2)	
Lymph node yield					
Mean ± SD	15.8 ± 6.9	15.6 ± 4.5	15.7 ± 5.6	15.7 ± 5.6	0.989
yp TNM stage					0.455
I	5 (16.1)	8 (26.7)	2 (11.1)	15 (19.0)	
IIA	4 (12.9)	4 (13.3)	5 (27.8)	13 (16.5)	
IIB	11 (35.5)	10 (33.3)	4 (22.2)	25 (31.6)	
IIC	0 (0)	0 (0)	1 (5.6)	1 (1.3)	
IIIA	0 (0)	2 (6.7)	1 (5.6)	3 (3.8)	
IIIB	10 (32.3)	6 (20.0)	4 (22.2)	20 (25.3)	
IIIC	1 (3.2)	0 (0)	1 (5.6)	2 (2.5)	
Tumor regression					0.218
Grade 0	5 (16.1)	8 (26.7)	2 (11.1)	15 (19.0)	
Grade 1	9 (29.0)	8 (26.7)	6 (33.3)	23 (29.1)	
Grade 2	7 (22.6)	12 (40.0)	6 (33.3)	25 (31.6)	
Grade 3	10 (32.3)	2 (6.7)	4 (22.2)	16 (20.3)	

Continuous variables are presented as means ± standard deviation (SD) and medians with ranges. Categorical variables are reported as numbers (%) and were compared using the χ^2^ test or Fisher’s exact test when expected counts were small; tumor height is defined as the distance from the anal verge to the tumor. UFT: tegafur-uracil; 5-FU/LV: 5-fluorouracil/leucovorin; ECOG PS: Eastern Cooperative Oncology Group performance status; RT-OP: interval from end of radiotherapy to surgery; CEA: baseline carcinoembryonic antigen; LAR: low anterior resection; APR: abdominoperineal resection.

**Table 2:** Maximum acute toxicities by regimen during preoperative concurrent chemoradiotherapy.

Toxicity	Grade	UFT (n = 31)	Capecitabine (n = 30)	5-FU/LV (n = 18)	*p*-Value
Neutropenia					0.003 ^†^
	0	31 (100.0)	24 (80.0)	15 (83.3)	
	1	0 (0.0)	5 (16.7)	0 (0.0)	
	2	0 (0.0)	1 (3.3)	2 (11.1)	
	3	0 (0.0)	0 (0.0)	1 (5.6)	
Anemia					0.746
	0	21 (67.7)	17 (56.7)	12 (66.7)	
	1	5 (16.1)	8 (26.7)	3 (16.7)	
	2	4 (12.9)	4 (13.3)	1 (5.6)	
	3	0 (0.0)	1 (3.3)	1 (5.6)	
	4	1 (3.2)	0 (0.0)	1 (5.6)	
Thrombocytopenia					0.460
	0	29 (93.5)	27 (90.0)	18 (100.0)	
	1	1 (3.2)	3 (10.0)	0 (0.0)	
	2	1 (3.2)	0 (0.0)	0 (0.0)	
Creatinine increased					0.444
	0	28 (90.3)	28 (93.3)	18 (100.0)	
	1	3 (9.7)	2 (6.7)	0 (0.0)	
Diarrhea					0.083
	0	13 (41.9)	4 (13.3)	8 (44.4)	
	1	11 (35.5)	19 (63.3)	7 (38.9)	
	2	7 (22.6)	7 (23.3)	3 (16.7)	
Proctitis					0.004 ^‡^
	0	20 (64.5)	14 (46.7)	16 (88.9)	
	1	6 (19.4)	15 (50.0)	2 (11.1)	
	2	5 (16.1)	1 (3.3)	0 (0.0)	
Dermatitis					0.946
	0	18 (58.1)	21 (70.0)	12 (66.7)	
	1	9 (29.0)	6 (20.0)	4 (22.2)	
	2	3 (9.7)	3 (10.0)	2 (11.1)	
	3	1 (3.2)	0 (0.0)	0 (0.0)	
Cystitis					0.208
	0	26 (83.9)	29 (96.7)	17 (94.4)	
	1	5 (16.1)	1 (3.3)	1 (5.6)	
Pain					0.115
	0	15 (48.4)	22 (73.3)	13 (72.2)	
	1	8 (25.8)	6 (20.0)	5 (27.8)	
	2	7 (22.6)	2 (6.7)	0 (0.0)	
	3	1 (3.2)	0 (0.0)	0 (0.0)	

Categorical data are presented as numbers (%) and were compared using the χ^2^ test or Fisher’s exact test when expected counts were small; toxicity was graded according to the version 4.0 of the Common Toxicity Criteria for Adverse Events. UFT: tegafur-uracil; 5-FU/LV: 5-fluorouracil/leucovorin; ^†^, ^‡^ *p* < 0.0056 (Bonferroni-adjusted significance level).

**Table 3:** Odds ratios of pathologic complete response, T downstaging, and N downstaging.

Regimen	pCR				T Downstaging		N Downstaging	
	Crude OR (95% CI)	*p*	Adjusted OR ^†^ (95% CI)	*p*	Crude OR (95% CI)	*p*	Crude OR (95% CI)	***p***
UFT	1		1		1		1	
Capecitabine	1.58 (0.44–5.68)	0.481	1.21 (0.31–4.73)	0.788	2.52 (0.86–7.39)	0.093	4.48 (1.48–13.59)	0.008
5-FU/LV	0.65 (0.11–3.76)	0.630	0.46 (0.07–3.02)	0.421	2.30 (0.67–7.86)	0.184	3.43 (0.98–11.97)	0.053

pCR: pathologic complete response; OR: odds ratio; CI: confidence interval; UFT: tegafur-uracil; 5-FU/LV: 5-fluorouracil/leucovorin; ^†^ Adjusted ORs were calculated using multivariable logistic regression, adjusted for age, sex, clinical T stage, clinical N stage, and interval between radiotherapy and surgery.

## Data Availability

De-identified datasets may be available from the corresponding author upon reasonable request and with permission from the Institutional Review Board.

## Supplementary Materials

The following supporting information can be downloaded at: https://www.mdpi.com/article/10.3390/curroncol33020079/s1, Dose constraints for target volume and organs at risk used for radiotherapy planning.

## Abbreviations

The following abbreviations are used in this manuscript:

5-FU/LV	5-fluorouracil/leucovorin
UFT	Tegafur-uracil
CCRT	Concurrent chemoradiotherapy
pCR	Pathologic complete response
OS	Overall survival
RFS	Recurrence-free survival
CRC	Colorectal cancer
LARC	Locally advanced rectal cancer
TME	Total mesorectal excision
TNT	Total neoadjuvant therapy
CT	Computed tomography
MRI	Magnetic resonance imaging
ECOG	Eastern Cooperative Oncology Group
CBC	Complete blood count
CEA	Carcinoembryonic antigen
RT	Radiotherapy
GTV	Gross tumor volume
CTV	Clinical target volume
PTV	Planning target volume
IMRT	Intensity-modulated radiotherapy
LAR	Low anterior resection
APR	Abdominoperineal resection
TRG	Tumor regression grade
CTCAE	Common Toxicity Criteria for Adverse Events
SD	Standard deviation
ANOVA	Analysis of variance
OR	Odds ratio
CI	Confidence interval
