# Supplementary material for: Preoperative Chemoradiotherapy with Tegafur-Uracil, Capecitabine, or 5-Fluorouracil/Leucovorin for Rectal Cancer in an Asian Cohort: A Real-World Comparison from the Pre-TNT Era
Source: Curr Oncol. 2026 Jan 30;33(2):79. doi: 10.3390/curroncol33020079 (PMC12939673; doi:10.3390/curroncol33020079)
Supplement: Supplementary file 1 [file curroncol-33-00079-s001.zip › curroncol-4080761-supplementary.pdf]

Supplementary/ Radiotherapy planning constraints

Planning objectives for target volumes were as follows:

- PTV:
  - ✓ D95 ≥ 100% of prescribed dose
  - ✓ Dmin > 93% of prescribed dose
  - ✓ Dmax < 110% of prescribed dose

Organ-at-risk (OAR) dose-volume constraints were applied according to our institutional standards:

- Small bowel:
  - ✓ V40 Gy < 30%
  - ✓ V45 Gy < 195 cc
- Bladder:
  - ✓ V40 Gy < 40%
  - ✓ V50 Gy < 5%
- Femoral heads (each):
  - ✓ V45 Gy < 25%

Treatment plans were iteratively optimized to meet target coverage and OAR constraints whenever feasible; minor deviations were allowed only when necessary to maintain adequate PTV coverage.
